# Surveillance of *Candida* spp Bloodstream Infections: Epidemiological Trends and Risk Factors of Death in Two Mexican Tertiary Care Hospitals

**DOI:** 10.1371/journal.pone.0097325

**Published:** 2014-05-15

**Authors:** Dora E. Corzo-Leon, Tito Alvarado-Matute, Arnaldo L. Colombo, Patricia Cornejo-Juarez, Jorge Cortes, Juan I. Echevarria, Manuel Guzman-Blanco, Alejandro E. Macias, Marcio Nucci, Luis Ostrosky-Zeichner, Alfredo Ponce-de-Leon, Flavio Queiroz-Telles, Maria E. Santolaya, Luis Thompson-Moya, Iris N. Tiraboschi, Jeannete Zurita, Jose Sifuentes-Osornio

**Affiliations:** 1 Department of Medicine, Salvador Zubiran National Institute of Medical Sciences and Nutrition, Mexico City, Mexico; 2 Hospital Escuela, Tegucigalpa, Honduras; 3 Division of Infectious Diseases, Escola Paulista de Medicina, Universidade Federal de São Paulo, São Paulo, Brazil; 4 Division of Infectious Diseases, National Cancer Institute, Mexico City, Mexico; 5 Department of Internal Medicine, Universidad Nacional de Colombia, Bogota, Colombia; 6 Department of Medicine, Universidad Peruana Cayetano Heredia, Lima, Peru; 7 Infectious Diseases, Hospital Vargas de Caracas and Centro Medico de Caracas, Caracas, Venezuela; 8 University Hospital, Universidade Federal do Rio de Janeiro, Rio de Janeiro, Brazil; 9 Department of Medicine, Memorial Hermann Texas Medical Center, University of Texas Medical School at Houston, Houston, Texas, United States of America; 10 Hospital de Clinicas, Universidade Federal do Parana, Curitiba, Brazil; 11 Department of Pediatrics, Hospital Luis Calvo Mackenna, Faculty of Medicine, Universidad de Chile, Santiago, Chile; 12 Department of Medicine, Clinica Alemana, Universidad del Desarrollo, Santiago, Chile; 13 Infectious Diseases Unit, Hospital de Clinicas Jose de San Martin, Buenos Aires, Argentina; 14 Hospital Vozandes, Facultad de Medicina, Pontificia Universidad Catolica del Ecuador, Quito, Ecuador; University of Wisconsin Medical School, United States of America

## Abstract

**Introduction:**

Larger populations at risk, broader use of antibiotics and longer hospital stays have impacted on the incidence of *Candida* sp. bloodstream infections (CBSI).

**Objective:**

To determine clinical and epidemiologic characteristics of patients with CBSI in two tertiary care reference medical institutions in Mexico City.

**Design:**

Prospective and observational laboratory-based surveillance study conducted from 07/2008 to 06/2010.

**Methods:**

All patients with CBSI were included. Identification and antifungal susceptibility were performed using CLSI M27-A3 standard procedures. Frequencies, Mann-Whitney U test or T test were used as needed. Risk factors were determined with multivariable analysis and binary logistic regression analysis.

**Results:**

CBSI represented 3.8% of nosocomial bloodstream infections. Cumulative incidence was 2.8 per 1000 discharges (incidence rate: 0.38 per 1000 patient-days). *C. albicans* was the predominant species (46%), followed by *C. tropicalis* (26%). *C. glabrata* was isolated from patients with diabetes (50%), and elderly patients. Sixty-four patients (86%) received antifungals. Amphotericin-B deoxycholate (AmBD) was the most commonly used agent (66%). Overall mortality rate reached 46%, and risk factors for death were APACHE II score ≥16 (OR = 6.94, CI_95%_ = 2.34–20.58, p<0.0001), and liver disease (OR = 186.11, CI_95%_ = 7.61–4550.20, p = 0.001). Full susceptibility to fluconazole, AmBD and echinocandins among *C. albicans, C. tropicalis,* and *C. parapsilosis* was observed.

**Conclusions:**

The cumulative incidence rate in these centers was higher than other reports from tertiary care hospitals from Latin America. Knowledge of local epidemiologic patterns permits the design of more specific strategies for prevention and preemptive therapy of CBSI.

## Introduction

*Candida* bloodstream infections (CBSI) are the fourth cause of nosocomial bloodstream infections in the United States [1]–[3], and account for up to 10% of all bloodstream infections in hospitalized patients [4]–[6]. The crude mortality rate of CBSI is about 40% (range 5 to 70%) [1], [7]. Well recognized risk factors for invasive candidiasis are: critically ill patients in intensive care units, diabetes mellitus, immunosuppressive states, mechanical ventilation, neutropenia, recent surgical procedures and prematurity [2], [6], [8].

Surveillance programs for CBSI have provided data regarding the incidence and species distribution of *Candida* spp. across the world. In Latin America a recently published paper reported an incidence of 1.18 episodes of candidemia per 1,000 admissions, with *Candida albicans*, *C. tropicalis* and *C. parapsilosis* accounting for >80% of cases [9]. Reports of CBSI from Mexican institutions are scarce and necessary [10], [11]. The objective of this study was to describe the incidence rate, the epidemiology of candidemia, and to assess factors associated with death among patients with CBSI hospitalized in two referral, tertiary care, medical institutions located in Mexico City.

## Materials and Methods

### Design, Patient Selection and Data Collection

We conducted a laboratory-based survey of CBSI in two tertiary care hospitals in Mexico, between July 2008 and June 2010: the Salvador Zubiran National Institute of Medical Sciences and Nutrition (INCMNSZ) and the National Cancer Institute (INCAN). We included all hospitalized patients with at least one positive blood culture for *Candida* sp. Clinical and demographic data were collected (age, gender, co-morbidities, use of antimicrobials and antifungals in the 3-months period prior to CBSI, previous use of glucocorticoids and chemotherapy within the 14 days before CBSI, neutropenia, invasive mechanical ventilation, total parenteral nutrition, fever, septic shock, APACHE II, and Karnosky score +/− 72 hours of the date of the incident CBSI)**.** Patients were followed up for 30 days after the CSBI episode or until death. A central venous catheter (CVC) related infection was defined as growth of >15 CFU from a 5-cm catheter tip by semi-quantitative (roll-plate) culture and a positive blood culture [12].

#### Ethics statement

This study was reviewed and approved by the local IRB's [Comisión de Ética en Investigación (Commission of Ethics in Research) at INCMNSZ and Comisión de Investigación y Bioética (Commission of Research and Bioethics) at INCAN]. Informed consent was not requested to the patients. The IRB's waived the need for written informed consent from the participants, because of the observational nature of the study. The investigators did not intervene in the standard of care. Patient records/information was kept confidential and all identifiers were erased prior to analysis.

### Microbiological Procedures

All *Candida* isolates were cultured in Sabouraud cefoperazone media, underwent germ tube testing, and identified using Ybc-Vitek (bioMerieux, Lyon, France). After identification, antifungal susceptibility testing was performed in all isolates for amphotericin B deoxycholate (AmBD), 5-flucytosine, voriconazole, posaconazole, fluconazole, caspofungin, micafungin and anidulafungin, following the standardized recommendations of CLSI M27-A3 for broth microdilution method [13].

### Statistical analysis

The incidence of CBSI was expressed in episodes per 1000-discharges and per 1000 patients-day. Categorical variables were analyzed by Pearson chi-square test or Fisher's exact test, as appropriate, and continuous variables by the Mann-Whitney U test. The *p* value ≤0.05 was considered statistically significant. For the assessment of factors associated with 30-day mortality we compared patients who survived with those who died. Variables with a *p* value ≤0.1 by univariate analysis were included in a multivariate analysis using a logistic regression model. Statistical analysis was performed using SPSS 20.0 software.

## Results

Data provided in this study came from hospital-based populations from two tertiary care medical centers, which are open-access national referral institutions. Populations attended in both institutions are mainly complex medical and surgical problems with variable degrees of immunosuppression (cancer, rheumatic diseases, HIV, diabetes mellitus). During the study period, there were 25,857 hospital discharges (24,986 from general wards and 1,295 from ICU) and 24,110 blood cultures were processed for 10,045 patients (mean 2.4 blood cultures per patient), 4,542 blood cultures (19%) from 1,938 patients were positive. Among these 1,938 episodes, 74 proved to be cases of CBSI (3.8%). The incidence rate of CBSI was 2.8 cases per 1000 discharges and 0.38 cases per 1,000 patients/day. The incidence in the ICU was 17 cases per 1000 discharges and 2.2 cases per 1,000 patients/day, while in general wards the incidence was 2 cases per 1,000 discharges and 0.28 cases per 1,000 patients/day.

The mean age was 46 years (±18), 29 patients (39%) had been hospitalized within the previous three months of the CBSI episode, and the median duration of hospitalization before the event was 16 days (0–149 days). At the occurrence of CBSI, 28 patients (38%) were on invasive mechanical ventilation (IMV), and 57 (77%) had received ≥2 types of antibiotics during the previous two weeks. Sixty-nine patients had a CVC, which was removed, in 41 of them, and the tips were sent for culture; only 14 of them (34%) had a positive culture. The other 28 CVCs were not removed. Other clinical characteristics are described in Table 1.

**Table 1:** Clinical and demographic characteristics of patients who survived or died within 30 days after candidemia.

Characteristics	Alive (n = 40)	Dead (n = 34)	All (n = 74)	P
Mean age (Years) ± s.d.^a^	48±17	45±19	46±18	0.26
Cancer – n (%)	24 (60)	18 (53)	42 (56.8)	0.54
Liver disease	2 (5)	7 (20.6)	9 (12)	0.07
Acute kidney injury	15 (37)	21 (62)	36 (48)	0.06
Chronic kidney disease	5 (12.5)	10 (29.4)	15 (20.3)	0.088
All types of transplant	1 (2.5)	5 (14.7)	6 (8.1)	0.08
Chemotherapy	9 (22.5)	12 (35.3)	21(28)	0.32
Surgery	24 (60)	12 (35)	36 (49)	0.03
Cardiovascular disease	11 (28)	14 (41)	25 (34)	0.23
Diabetes mellitus	8 (20)	7 (21)	15 (20)	1
Autoimmune disease	7 (18)	7 (21)	14 (19)	0.77
Prior use of glucocorticoids	11 (27.5)	19 (55.9)	30 (40.5)	0.018
Neutropenia	9 (22.5)	12 (35.3)	21 (28)	0.3
TPN^b^	10 (25)	9 (26.5)	19 (25.7)	0.88
Mechanical ventilation	8 (20)	20 (58.8)	28 (37.8)	0.001
Antibiotics (more than 4 types)^c^	23 (57.5)	25 (73.5)	48 (64.9)	0.22
Fever	37 (92.5)	33 (97)	70 (94.6)	0.62
Severe sepsis	9 (22.5)	23 (67.6)	32 (43)	<0.0001
Septic shock	2 (5)	17 (50)	19 (25.7)	<0.0001
Candida deep infection	4 (10)	8 (23.5)	12 (16.2)	0.2
APACHE II Score	12 (3–25)	25 (12–36)	18 (3–36)	< 0.0001
Delayed start of treatment (days)	1 (2–6)	1 (0–8)	1 (2–8)	0.79
Duration of antifungal therapy	15 (1–55)	6 (1–41)	13 (1–55)	0.004
Untreated patients	3 (7.5)	7 (20)	10 (13.5)	0.17

a s.d., Standard deviation; ^b^TPN, Total parenteral nutrition; ^c^families of antibiotics were: broad–spectrum penicillins, broad-spectrum cephalosporins, carbapenems, aminoglycosides, glycopeptides, linezolid or quinolones.

*C. albicans* was the most common species (n = 34, 46%). Among the non-*albicans* species, C. *tropicalis* was the most frequent (n = 19, 26%), followed by *C. glabrata* (n = 10, 13.5%), *C. parapsilosis* (n = 4, 5%), *C. krusei* (n = 4, 5%), *C. guilliermondii* (n = 2, 3%) and *C. lipolytica* (n = 1, 1%). *C. glabrata* was isolated most frequently in elderly patients (65±13 vs 44±17 years, p<0.0001) and in those with diabetes mellitus (50 vs 15%, p = 0.024). *C. tropicalis* was more commonly observed in patients with hematologic cancer and severe neutropenia (53% vs. 20%, p = 0.01).

Nineteen patients (26%) received antifungals prior to the CBSI (fluconazole [n = 10], AmBD [n = 8] and voriconazole [n = 1]). They received any of these drugs for up to 12 days (median of 2 d) because of prophylaxis, prior fungal infection or preemptive therapy. Sixty-four patients (86%) received antifungal therapy to treat the episode of CBSI, AmBD was the most commonly used agent (n = 42, 66%), followed by echinocandins (n = 13, 20%), and fluconazole (n = 8, 13%). One patient received a combination of fluconazole and AmBD (1%). Twenty-nine of the 64 patients who received therapy died (45.3%), 17 of them within the first 7 days of antifungal treatment. Ten patients did not receive antifungal treatment, five died the day that blood samples were drawn, two died within the next three days of isolation, and the other three asked for voluntary discharge.

Overall, 28 of the 74 patients (38%) died within 15 days after diagnosis of CBSI and 34 (46%) within 30 days. There were no differences in mortality between the different species of *Candida*. Likewise, no differences in mortality rates were observed among ICU and non-ICU patients or types of antifungal therapy. By contrast, variables associated with higher mortality rates by univariate analysis were: prior use of glucocorticoids, liver disease, removal of CVC, IMV, low Karnofsky score, severe sepsis, septic shock, and APACHE II score ≥16 (Table 1).

Prognostic factors associated with 30-day mortality assessed by univariate and multivariate analysis are shown in Table 2. In multivariate analysis, independent variables associated with death were liver disease, and APACHE II score ≥16. Removal of CVC at anytime was associated with a favorable outcome in the univariate analysis, but it was not confirmed in the multivariate analysis. (Table 2).

**Table 2:** Risk factors for death: Cox proportional-hazards regression model.

Characteristic	Univariate HR (IC 95%)	P	Multivariate* HR (IC 95%)	P
APACHE II Score^a^	6.94 (2.34–20.58)	<0.0001	12.13 (1.89–77.5)	0.008
Liver disease	11.25 (1.32–95.37)	0.026	186.11 (7.61–4550.20)	0.001
Invasive mechanical ventilation	2.40 (0.91–6.29)	0.074		
CVC removal ^b^	0.26 (0.08–0.81)	0.020		
Prior use of steroid	0.42 (0.16–1.08)	0.073		

a ≥16 points; ^b^ CVC, Central venous catheter.

Antifungal susceptibility data of 68 isolates were available (Table 3). All isolates were susceptible to echinocandins. For micafungin and anidulafungin, the MIC90 value was ≤0.06 mcg/ml, and for caspofungin was ≤0.25 mcg/ml. The MIC90 of voriconazole and posaconazole for all species was ≤0.125 mcg/ml. All isolates of *C. albicans, C. parapsilosis* and *C. tropicalis* were susceptible to fluconazole using CLSI M27-A3 guidelines. The MIC90 of fluconazole for *C. albicans* and *C. tropicalis* was 1 mcg/ml, and for *C. parapsilosis* 0.5 mcg/ml. Two of nine *C. glabrata* isolates tested, showed high MICs values for fluconazole, one showed resistance to all azoles, and the other one exhibited a high MIC value only for fluconazole. The two isolates of *C. guilliermondii* were susceptible to fluconazole (2 and 4 mcg/ml), and one *C. lipolytica* showed an MIC value of 2 mcg/mL for fluconazole (Table 3).

**Table 3:** Activity of antifungal agents among *Candida* species isolated during this study using CLSI M27-A3 BMD methods (n = 67)^a,,b^.

Species (No. tested)	Antifungal Agent	MIC (mcg/ml)			% Resistance
		Range	MIC^50^	MIC^90^	
*C. albicans* (n = 32)	Amphotericin	0.125–2	0.5	1	
	Anidulafungin ^c^	0.015–0.06	0.015	0.03	0
	Caspofungin^ c^	0.015–0.06	0.03	0.06	0
	Micafungin ^c^	0.015–0.03	0.015	0.015	0
	Fluconazole	0.125–1	0.25	1	0
	Posaconazole	0.03–0.06	0.03	0.03	0
	Voriconazole	0.03–1	0.03	0.03	0
	Flucytosine	0.25–4	0.125	0.5	0
*C. tropicalis* (n = 18)	Amphotericin	0.25–2	0.5	1	
	Anidulafungin ^c^	0.015–0.06	0.015	0.03	0
	Caspofungin ^c^	0.015–0.25	0.06	0.125	0
	Micafungin ^c^	0.015–0.03	0.015	0.03	0
	Fluconazole	0.125–2	0.25	1	0
	Posaconazole	0.03–0.06	0.03	0.03	0
	Voriconazole	0.03–0.25	0.03	0.06	0
	Flucytosine	0.125–64	0.125	0.5	0
*C. glabrata* (n = 9) ^d^	Amphotericin	0.125–2	0.5	1	
	Anidulafungin ^c^	0.015–0.06	0.015	0.03	0
	Caspofungin ^c^	0.06–0.125	0.125	0.125	0
	Micafungin ^c^	0.015–0.06	0.015	0.015	0
	Fluconazole	1–64	4	16	11
	Posaconazole	0.06–2	0.25	0.25	11
	Voriconazole	0.06–4	0.25	0.25	11
	Flucytosine	0.125–0.5	0.125	0.25	0
*C. parapsilosis* (n = 4)	Amphotericin	0.25–0.5	0.5	0.5	
	Anidulafungin ^c^	0.06–1	0.5	1	0
	Caspofungin ^c^	0.25	0.25	0.25	0
	Micafungin ^c^	0.25–1	0.5	1	0
	Fluconazole	0.125–0.5	0.25	0.5	0
	Posaconazole	0.03	0.03	0.03	0
	Voriconazole	0.03	0.03	0.03	0
	Flucytosine	0.125	0.125	0.125	0
*C. krusei* (n = 2)	Amphotericin	1–2	1	2	
	Anidulafungin ^c^	0.06–0.125	0.06	0.125	0
	Caspofungin ^c^	0.25–0.5	0.25	0.5	0
	Micafungin ^c^	0.06	0.06	0.06	0
	Fluconazole	16–32	16	32	
	Posaconazole	0.125	0.125	0.125	0
	Voriconazole	0.25	0.25	0.25	0
	Flucytosine	16–32	16	32	100

a. MICs determined based on CLSI M27-A3; b. The susceptibilities of 3 species are not shown (2-*C. guilliermondii*, and 1-*C. lipolytica*); c. *Non-resistant iso*lates we*re identified* for echinocandins; d. One *C. glabrata* isolate showed dose-dependent susceptibility (MIC = 16 mcg/ml) and susceptibility to voriconazole and posaconazole. One *C. glabrata* isolate was resistant to fluconazole (MIC = 64 mcg/ml), voriconazole (MIC = 4 mcg/ml) and posaconazole (MIC = 2 mcg/ml).

## Discussion

This is the first surveillance study of CBSI performed in Mexico in which incidence rates, species distribution, antifungal therapy and predictors of outcome were analyzed. We observed a high incidence of CBSI. Common risk factors identified previously were confirmed. A high crude mortality rate was observed which was associated with high APACHE II scores and other severe conditions. All clinical isolates included in this report were fully susceptible to echinocandins.

The cumulated incidence (2.8 cases per 1000 discharges) and incidence rate (0.38 cases per 1000 patient-days) reported in this study are higher than the ones from other recent reports. Studies done in other tertiary care hospitals in the European Community and the USA have shown cumulated incidence between 0.17 and 1 case per 1000 discharges [14]. Nucci et al. recently reported lower incidence rates (1.18 cases per 1,000 discharges and 0.23 cases per 1,000 patients-day) in 21 tertiary care hospitals in 7 countries in South and Central America [9].

The incidence rate in this study was 0.38 cases per 1000 patient-days, which is similar to the incidence rate reported in the nationwide sentinel surveillance study done in Brazil (0.37 cases per 1000 patients/day) during last decade [5], [7]. In Switzerland, after a 10-year surveillance in tertiary care centers, the overall incidence rate was 0.049 cases per 1,000 patient-days [14]. Including the findings of this study, the incidence rate seen in tertiary care hospitals in Latin America is almost 100 times greater than those seen in the European Community. In summary, we observed a higher cumulated incidence, and a higher incidence rate than in other regions, including Latin American countries.

*C. albicans* continues to be the most common species causing CBSI worldwide (»40% to 60% of cases) and it was the main agent (46%) in this study as well, even though it has been well documented that since the introduction of second-generation azoles there has been a steady increase in the isolation of non-albicans species [15].

In our study, *C. tropicalis* was the most common non-*albicans* agent (26%) followed by *C. glabrata* (13.5%), whereas *C. parapsilosis* was an uncommon pathogen (5%). In the USA, the most common species, after *C. albicans*, has been *C. glabrata* (10 to 21%) [1], [16]. *C. parapsilosis* is one of the most common isolates in Latin America (20%), in contrast, *C. glabrata* has been uncommon in Latin American reports (<6%) [5], [16].

Different distribution patterns could be explained by three main factors, the intensity of use of fluconazole for prophylaxis, the age of the population at risk, and infection control practices. Prophylaxis with fluconazole has been associated with a decrease in the rate of CBSI among patients with hematologic malignancies; however, its use favors the selection of resistant species such as *C. glabrata* [8], [17], [18]. In the USA, prophylaxis with fluconazole is used more extensively than in Latin America, which may account for the difference in patterns. The second point has been recently raised since *C. parapsilosis* is the principal mucosal colonizer in the newborn population, whereas *C. glabrata* is more common in elderly patients [1], [19]. Our study included adult population (mean age 46±18 years), whereas most of the Latin American studies have included newborn, pediatric, and adult populations [1], [10], [19]. Finally, there are strong differences in the infection control practices in the world and *C. parapsilosis* has been associated with outbreaks of CVC related CBSI. Therefore, high rates of *C. parapsilosis* recovery could indicate careless CVC handling in many settings [10], [15], [20], [21]. The low prevalence of *C. parapsilosis* among the populations included in this study confirms the strict policy of CVC care, as seen in previous reports from both participant institutions [22], [23].

Liver disease and APACHE II score ≥16 were the major risk factors of mortality. CBSI has been associated with the development of septic shock in 23% of the cases [4], [24], [25]. Increased mortality rates have been noticed in patients with CBSI who did not receive empirical/preemptive therapy within the first 24 hrs, mainly in patients with septic shock [4], [26], [27]. We observed a high mortality rate within the first 7 days, which may confirm indirectly the need of early and preemptive treatment in critically ill patients, as reported elsewhere in hematopoietic stem cell transplant recipients [28], [29]. The majority of those who received treatment for less than 6 days were critically ill and died soon after CBSI diagnosis, which could have limited the efficacy of antifungals.In our study, not removing the CVC was not associated with a poorer outcome by multivariate analysis. However, our number of patients is small and therefore, a stronger recommendation regarding CVC management is not warranted [9], [30], [31].

Most of the clinical isolates were susceptible to all antifungal agents. In Latin America, AmBD and fluconazole are broadly used, with less extended use of echinocandins. The less extended use of echinocandins is mostly due to lack of access and high prices to these agents [30], [32]. International studies have reported resistance to fluconazole in less than 1.5% of *C. albicans, C. tropicalis* and *C. parapsilosis* isolates [33]. In this study, *C. albicans, C. tropicalis* and *C. parapsilosis* were 100% susceptible to all kind of antifungal agents. However, potential resistant species were identified in 21% of our study population (*C. glabrata* 10, *C. krusei* 4, *C. guilliermondii* 2 and *C. lipolytica* 1). The main resistant species, reported worldwide, is *C. glabrata*, which was not as common as *C. albicans*, and *C. tropicalis* in our study. Usually, *C. glabrata* is associated with approximately 30% of resistance to fluconazole [33], [34]; in this study, the rate of resistance of *C. glabrata* was lower than prior reports.

Despite the susceptibility patterns described in our report, continuous surveillance programs are needed in order to identify possible changes in the species distribution and antifungal susceptibility patterns of yeasts, particularly in Latin American hospitals [21], [33].

This study has several limitations such as the low number of cases, the restricted geographic region where the study was performed, and the type and number of institutions included. Both institutions are open-access tertiary care referral centers, which attend only adult patients. These characteristics may limit the generalization of our findings to other geographic zones in Mexico and Latin America, and also, cannot be applied to other clinical settings, such as pediatric and newborn medical units. Finally, the number of isolates included could underestimate the antifungal resistance rate among the population studied.

In conclusion, in this study we described a higher incidence of CBSI than that of others countries, as well as differences in the epidemiology of CBSI in comparison with previous reports. The reasons of these differences are not clear. Nevertheless, longer and more comprehensive surveillance programs will allow us to have a better understanding of the epidemiology and susceptibility patterns of CBSI in limited-resource countries.
